# Hyper-induction of IL-6 after TLR1/2 stimulation in calves with bovine respiratory disease

**DOI:** 10.1371/journal.pone.0309964

**Published:** 2024-11-14

**Authors:** Cian Reid, John Donlon, Aude Remot, Emer Kennedy, Giovanna De Matteis, Cliona O’Farrelly, Conor McAloon, Kieran G. Meade

**Affiliations:** 1 Animal & Bioscience Research Department, Animal & Grassland Research and Innovation Centre, Teagasc, Grange, Co Meath, Ireland; 2 School of Biochemistry and Immunology, Trinity College Dublin, Dublin 2, Ireland; 3 School of Veterinary Medicine, University College Dublin, Dublin, Ireland; 4 INRAE, Université de Tours, Nouzilly, France; 5 Teagasc, Animal & Grassland Research and Innovation Centre, Moorepark, Fermoy, Co. Cork, Ireland; 6 Council for Agricultural Research and Economics, Research Centre for Animal Production and Aquaculture, CREA-ZA, Italy; 7 School of Medicine, Trinity College Dublin, Dublin 2, Ireland; 8 School of Agriculture and Food Science, University College Dublin, Belfield, Dublin 4 Ireland; 9 Conway Institute of Biomolecular and Biomedical Research, University College Dublin, Belfield, Dublin 4, Ireland; 10 Institute of Food and Health, University College Dublin, Belfield, Dublin 4, Ireland; Kerman University of Medical Sciences, ISLAMIC REPUBLIC OF IRAN

## Abstract

Bovine respiratory disease (BRD) is a leading cause of mortality and compromised welfare in bovines. It is a polymicrobial syndrome resulting from a complex interplay of viral and bacterial pathogens with environmental factors. Despite the availability of vaccines, incidence and severity in young calves remains unabated. A more precise analysis of host innate immune responses during infection will identify improved diagnostic and prognostic biomarkers for early intervention and targeted treatments to prevent severe disease and loss of production efficiency. Here, we investigate hematological and innate immune responses using standardized *ex-vivo* whole blood assays in calves diagnosed with BRD. A total of 65 calves were recruited for this study, all between 2–8 weeks of age with 28 diagnosed with BRD by a thoracic ultrasonography score (TUS) and 19 by Wisconsin health score (WHS) and all data compared to 22 healthy controls from the same 9 study farms. Haematology revealed circulating immune cell populations were similar in both TUS positive and WHS positive calves compared to healthy controls. Gene expression analysis of 48 innate immune signalling genes in whole blood stimulated with TLR ligands was completed in a subset of calves. TLR1/2 stimulation with Pam3CSK4 showed a decreased pattern of expression in IL-1 and inflammasome related genes in addition to chemokine genes in calves with BRD. In response to TLR ligands LPS, Pam3CSK4 and R848, protein analysis of supernatant collected from all calves with BRD revealed significantly increased IL-6, but not IL-1β or IL-8, compared to healthy controls. This hyper-induction of IL-6 was observed most significantly in response to TLR1/2 stimulation in TUS positive calves. ROC analysis identified this induced IL-6 response to TLR1/2 stimulation as a potential diagnostic for BRD with a 74% true positive and 5% false positive detection rate for an IL-6 concentration >1780pg/mL. Overall, these results show altered immune responses specifically upon TLR1/2 activation is associated with BRD pathology which may contribute to disease progression. We have also identified induced IL-6 as a potentially informative biomarker for improved early intervention strategies for BRD.

## Introduction

Bovine respiratory disease (BRD) is a multifactorial syndrome, caused by both viral and bacterial agents infecting the upper and lower respiratory tract. It is a leading cause of mortality in calves in Ireland, accounting for 34% of deaths in calves from 1–5 months of age submitted for post mortem examination [1]. BRD also negatively affects the sustainability of the animal production sector causing estimated economic losses of €576 million a year in Europe [2]. Susceptibility to BRD is heavily influenced by stressors, including poor ventilation and transportation which combine to weaken the developing immune system of young calves [3]. Therefore, immune biomarkers may enable early detection of BRD, improve accuracy of diagnosis and prognosis, and in turn reduce disease burden and current dependency on antibiotic usage. Understanding the mechanisms of BRD infection could also identify targets for future immunotherapies. However, the innate immune mechanisms leading to BRD severity and subsequent pathology remain elusive.

Typically, BRD pathology develops after a calf with an underdeveloped or suppressed immune system is exposed to a viral pathogen which infects the respiratory tract including bovine respiratory syncytial virus (BRSV), bovine viral diarrhea virus (BVDV), bovine herpes virus 1 (BHV-1) or bovine parainfluenza virus 3 (BPIV-3). The viral pathogen then further perturbs host homeostasis in the lungs, resulting in growth of secondary bacterial pathogens such as *Mycoplasma bovis*, *Mannheimia haemolytica*, *Pasturella multocida* and *Histophilus somni*. This development of a polymicrobial syndrome can result in subsequent progression to clinical pneumonia [3]. However, stressors may also alter the relative abundance of bacterial taxa thereby contributing to pathology even in the absence of viral infection [4]. Widespread vaccination against BRD viral and bacterial pathogens has failed to substantially reduce disease burden and mortality, particularly young calves of the pre-weaning age [5]. This may be due to the underdeveloped adaptive immune responses in young calves which is reflected in low numbers of circulating T and B lymphocytes and in limited endogenous antibody production. Therefore, innate immune mechanisms are likely a key determinant of susceptibility and the extent of pathology which results from BRD infection.

Currently, on-farm clinical assessments of symptoms related to pathology are most commonly used to diagnose BRD. These include the Wisconsin health score (WHS), which assigns scores to symptoms associated with BRD i.e., high temperature, persistent cough, eye and nasal discharge [6]. However, previous studies have estimated that clinically scoring respiratory symptoms using WHS can fail to detect 20–51% of BRD infections identified by lung lesions post-mortem, which is most likely due to variation in lesion location and the variable severity of infection [7, 8]. In recognition of these limitations, ultrasound technology is often used to score the severity of lung lesions associated with BRD, known as a thoracic lung ultrasonography score (TUS). TUS has been reported as a more sensitive measure of BRD and can more accurately identify animals with sub-clinical disease [9, 10]. Therefore, the combined use of TUS and WHS may increase the precision of overall disease diagnosis. A positive case of BRD diagnosed by WHS but with no lung lesions present (as evaluated by TUS) may reflect a case of upper-respiratory tract infection. Alternatively, a positive case diagnosed by TUS but negative by WHS may identify lower-respiratory tract infection which is not yet causing hallmark clinical symptoms. A case classified as positive by both techniques is thought to identify a more severe or advanced case of BRD. It is our contention that the collection of reproducible innate immune phenotypes in tandem with clinical assessments could potentially offer new insights into disease pathogenesis.

Respiratory epithelial cells are usually the first cells to encounter inhaled pathogens and *in vitro* studies have shown viral pathogens BVDV and BRSV inhibit the host-mediated expression of type I interferon cytokines, thereby limiting the efficacy of the anti-viral immune response [11–13]. Furthermore, bovine bronchial epithelial cells stimulated with *M*. *haemolytica* or BHV-1 expressed high TNF-α, IL-6 and IL-8 with coinfection of both pathogens, exacerbating the inflammatory response [11, 14]. Cattle experimentally infected with highly virulent BVDV compared to low virulent BVDV exhibited decreased expression of interferon-stimulated genes (ISGs), elucidating a possible mechanism of immune suppression potentially contributing to the development of severe disease [15]. Systemic profiling of pro-inflammatory cytokine responses has also been performed in *in vivo* experimentally infected animals [16]. However, less is known about the innate immune responses associated with susceptibility and pathology in calves with naturally acquired infection.

Studies in naturally BRD infected animals have also detected significantly elevated pro-inflammatory cytokines, both locally and systemically in the absence of stimulation that are indicative of clinical disease status [17, 18]. However, studies in human subjects have found that *ex-vivo* stimulation of the immune system can be more robust in the identification of dysfunctional immune response mechanisms during disease which may not be apparent in the non-induced state. In humans admitted to hospital with COVID-19, for example, impaired IL-1β responses from whole blood stimulated with LPS *ex-vivo* was associated with future severity of disease [19]. Another study using the same approach found that defective type I IFN responses to viral ligand R848 was associated with disease severity [20]. Similar approaches in BRD infected cattle could elucidate if dysfunctional immune responses are associated with BRD infection. However, standardized innate immune response phenotyping has not been carried out in calves naturally infected with BRD. Therefore, the objective of this study was to perform whole blood stimulations with pathogen-relevant Toll-like receptor (TLR) ligands to investigate the specific induced innate immune responses of calves naturally infected with BRD.

## Materials and methods

### Ethical statement

All experimental procedures were approved by the Teagasc Ethics Committee and University College Dublin and were conducted under the experimental licenses (AE19132/P100 and AE19132/P099, AE19132/P105 and V016/2020Q1) from the Health Products Regulatory Authority in accordance with the Cruelty to Animals Act (Ireland 1876) and the European Community Directive 2010/63/EU.

### Animal resources

A total of 65 calves were sampled from 9 commercial farms based around Ireland that had been identified as having recurrent BRD incidence from previous years. They were Spring-born, mixed-breed, housed inside and aged between 2–8 weeks old. For every calf diagnosed with BRD, a healthy control was sampled from the same farm. Further details about each calf can been seen in S1 Table.

### Haematology

Haematology profiles were established from blood collected in 6 mL EDTA coated vacutainers processed using the ADVIA 2120 haematology analyzer system to acquire total lymphocyte, monocyte, neutrophil, basophil and eosinophil counts.

### *ImmunoChek—ex-vivo* whole blood stimulations

Whole blood innate immune phenotyping assay *ImmunoChek* was carried out as described in [21]. Briefly, 1mL of whole blood collected in sodium heparin tubes from the calves was added to each of the following prefilled culture tubes (Sarstedt); 2mL of media (unstimulated), 2μg/mL of LPS (Serotype O111:B4, Merck) in 2 mL of media, 1μg/mL of Pam3CysSerLys4 (Pam3CSK4) [Invivogen] in 2mL of media and 0.2μg/mL of imidazoquinoline R848 (Invivogen) in 2mL of media. Media was prepared using RPMI media supplemented with 50μg/mL streptomycin and 2.5μg/mL amphotericin B (ThermoFisher Scientific). Culture tubes were then incubated at 38.5°C for 24 hours for stimulations. Supernatants were collected and stored at -20°C while cell pellets were snap frozen in dry ice and stored at -80°C.

### RNA extraction from *ImmunoChek* stimulations, cDNA synthesis and gene expression analysis

A subset of animals based on TUS positivity were selected for RNA extraction and gene expression analysis after 4 hour (IDs: 31612, 52802, 55470, 95656, 35444, 32841, 65653, 32783, 22816, 12823, 41341, 61376. See S1 Table for details) and 24 hour stimulations (IDs: 65653, 64695, 53720, 94731, 31961, 22816, 52802, 23734, 51334, 71385, 64712. See S1 Table for details). RNA extraction was carried out using a combination of Trizol method and the RNeasy Mini Kit Qiagen as outlined in [22]. Briefly, 800μL of Trizol was added to frozen cell pellets of post stimulation while defrosting. Chloroform was added and then centrifuged 12,000 *x g* for 15 minutes. Then, 800uL of 70% ethanol was added to the sample. This was then added to the RNeasy column in which the rest of the procedure was carried out per manufacturer instructions. RNA quantity was calculated using a nanodrop. RNA quality was calculated using the RNA Nano kit (Agilent Technologies) and a bioanalyzer instrument. The collected RNA was then diluted to equal concentrations and converted to cDNA using the High-Capacity cDNA Reverse Transcriptase kit, as per manufacturer instructions (Thermofisher).

Gene expression analysis was blindly carried out with cDNA using Biomark HD (Fluidigm) in a 48x48 well plate, as per manufacturer instructions. Primer sequences are shown in S3 Table (all designed to work with an annealing temperature of 60°C). Briefly, 1 μL of 96 pooled primers (100μM) were added to 1.25 μL of 1/10 diluted cDNA samples in a 96 well plate. Pre-amplification master mix was added to each cDNA sample. Target sites of the 96 primers were then pre-amplified by 14 cycles of thermocycling (hold at 95°C for 2 minutes, denaturation at 95°C for 15 seconds, annealing at 60°C for 4 minutes, hold at 4°C). Next, 48 μL of 4 units per μL exonuclease was added to each sample and added to one thermocycling run of 30 minutes at 37°C, 15 minutes at 80°C and then cooled to 4°C to remove primers from samples. Primers-free samples were diluted 5 times in TE buffer. Then, 2.75 μL of EvaGreen sample pre-mix is added to 2.25 μL of exonuclease treated sample and added to the sample inlets in the Fluidigm chip. Each primer (0.25 μL, 100μM) is mixed with 2.5 μL of assay loading reagent and 2.25 μL of DNA suspension buffer and added to assay inlets in Fluidigm chip. Fluidigm chip was inserted into Biomark instrument and ran at appropriate settings for gene expression analysis according to manufacturer instructions. Fluidigm Real-Time qPCR software was used to calculate the Ct values for each gene in each sample. Ct values were then normalized to three (*GAPDH*, *PPIA* and *ACTB*) normalizer genes to calculate ΔCt values [23]. For the unstimulated expression, these values were centered to the mean and scaled by unit variance ((mean-ΔCt)/standard deviation) for use in principal component analysis (PCA), heatmaps and radar plots. For PAMP induced responses, fold changes were calculated by normalizing the unstimulated expression using the ΔΔCt method [22]. The fold changes were then logged (log_2_(FC)) and mean centered and scaled by unit variance ((x-Log2FC)/standard deviation) for use in PCA and to generate heatmaps.

### Gene expression scores

As performed previously [22], gene expression scores were calculated using a z scoring system and has been previously used on gene expression results from *ex-vivo* blood stimulations in humans [24]. Firstly, genes were organized into groups based on signalling pathways and function. Z scores were then calculated for each gene by mean centering and scaling by unit variance, using the ΔCt value for unstimulated expression ((mean-ΔCt)/standard deviation), and Log2FC for PAMP stimulated expression ((x-Log2FC)/standard deviation). The average z score for each gene within a group was then calculated, giving each calf a gene expression score for that group.

### Thoracic ultrasonography scoring (TUS) and Wisconsin health scoring (WHS)

Each calf was assessed using TUS and Wisconsin scoring to diagnose BRD. TUS involves using ultrasound to detect the presence of lesions associated with BRD. Primary lesions of interest were detected by presence of consolidation (S1A Fig), while a lung which is air filled produces a reverberation artefact (S1B Fig). All TUS was performed using a linear ultrasound probe set at a depth of 7cm and frequency of 7MHz (Easi-Scan Go, IMV Technology Ltd.). A 70% isopropyl alcohol solution was sprayed onto the unclipped hair on both sides of the thorax. The ultrasound probe was then used to scan each intercostal space dorsoventrally starting at the 10^th^ intercostal space on the left-hand side and moving cranially till the 2^nd^ intercostal space on the left-hand side. On the right hand-side a similar technique was used but the fore limb was drawn forward to allow for imaging of both the 1^st^ and 2^nd^ intercostal spaces. Each calf was then assigned a score between 0 and 5 based on the presence and extent of lesions detected on ultrasound [25]: 0 = normal aerated lung with no consolidation and one to few comet tail artifacts; 1 = diffuse small comet tail artifacts without consolidation; 2 = isolated patches of consolidation, 3 = consolidation of a full lung lobe, 4 = consolidation of 2 lung lobes, 5 = consolidation of 3 or more lung lobes.

The Wisconsin score was determined by scoring nasal and ocular discharge, ear position, presence of cough/response to a tracheal pinch and rectal temperature. Each of these components were assigned a score from 0–3 depending on the degree of deviation from normal with 0 being normal and 3 being severely abnormal. The component scores were then summed and total score ≥5 was considered diagnosis of BRD [26].

### ELISAs

Protein analysis in the supernatants of the *ex-vivo* whole blood stimulations was carried out by ELISA assays, using IL-1 beta Bovine Uncoated ELISA Kit (Thermofisher) to measure IL-1β levels and IL-6 Bovine Uncoated ELISA kit (Thermofisher) to measure IL-6 levels, as per manufacturer instructions, also used previously with *ImmunoChek* system [21]. IL-8 was also measured using ELISA as previously described [27].

### Statistical analysis

Statistical analysis and plot generation was done in R studio. A linear regression model correcting for age and sex was used to calculate the differences in hematological cell counts between controls and TUS positive animals, and controls and WHS positive animals. A linear regression model correcting for age and sex was also used to calculate the differences in unstimulated and PAMP stimulated protein expression of IL-6, IL-1β and IL-8 between controls and TUS positive animals, and controls and WHS positive animals. T-tests were carried out to test differences between groups for gene expression. False discovery rate (FDR) method of correction was used for *p* value adjustment, with a significance cut off point of *p* < 0.1. Unpaired t tests were used to calculate differences between groups for gene expression scores. All box and dot plots were made with ggplot2 package, PCA plots in Factoextra package, heatmaps with ComplexHeatmaps package and radar plots with fmsb package. ComplexHeatmaps package carries out hierarchical clustering using the k-means clustering method. Receiver operator characteristic (ROC) was carried out in R studio with pROC package. Tukey analysis was used to identify outliers in unstimulated protein expression in whole blood stimulations.

## Results

### Similar hematological profiles between controls and BRD diagnosed calves

**Fig 1:**
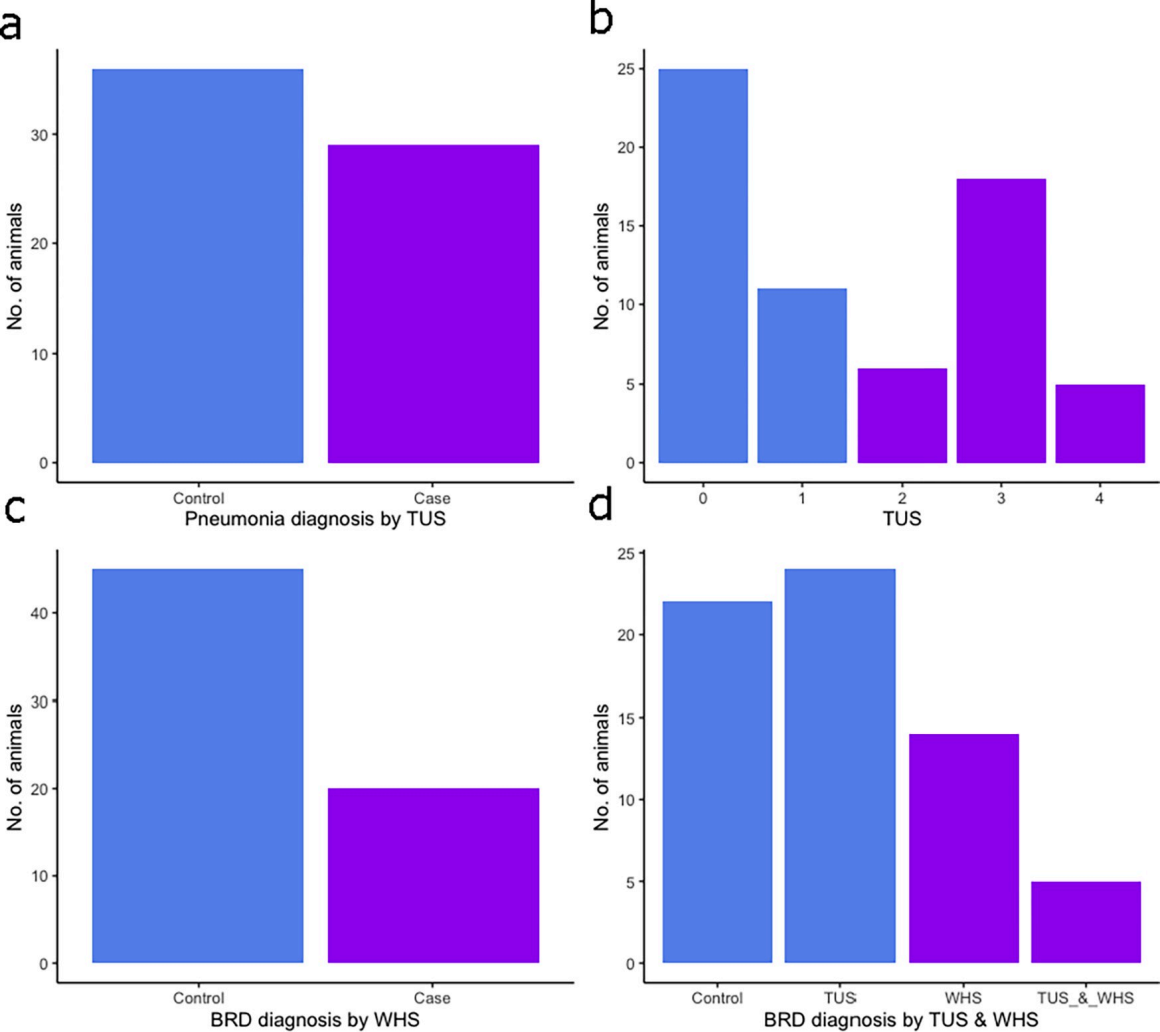
Calves diagnosed as a case of bovine respiratory disease (BRD) by thoracic lung ultrasonography scoring and Wisconsin health scoring (WHS). Histograms of the number of calves (n = 65) aged 2–8 weeks old (a) assigned controls and BRD cases by TUS, (b) assigned each score from TUS system (0–4), (c) assigned controls and BRD cases by WHS and (d) assigned controls from both TUS and WHS, assigned BRD case by TUS only, assigned BRD case from WHS, and assigned BRD case from both TUS and WHS.

**Fig 2:**
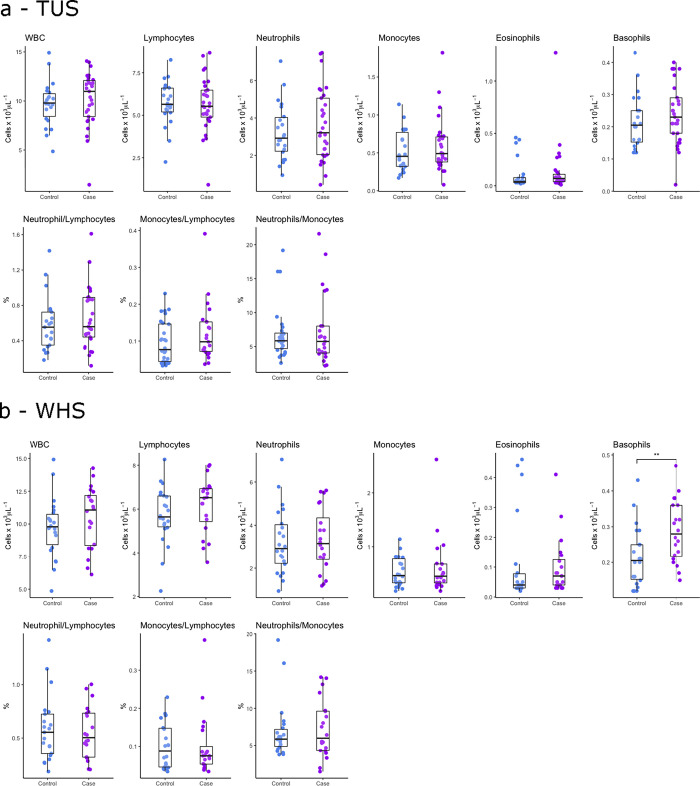
Increased basophils in blood of calves diagnosed with BRD by Wisconsin health scoring. Boxplots of the cell counts in blood of white blood cells (WBC), lymphocytes, neutrophils, monocytes, eosinophils, basophils, and ratio of neutrophil to lymphocytes, monocytes to lymphocytes and neutrophils to monocytes in healthy controls and (a) TUS positive and (b) WHS positive calves. The box represents the interquartile range, the whiskers the upper and lower limits and the dots representing each calf. Any calves outside the upper and lower limits considered outliers (Tukey outlier analysis). T tests were used to test for significant differences between cases and controls. *p<0.05, **p<0.01.

A total of 65 calves aged between 2–8 weeks old were recruited from farms (n = 9) with a high incidence of BRD in which thoracic lung ultrasonography scoring (TUS) and Wisconsin health scoring (WHS) were performed. Haematological analysis and 4 hour and 24-hour whole blood null, LPS, Pam3CSK4 and R848 stimulations were carried out using *ImmunoChek*. From these calves, 29 were diagnosed as positive for BRD by TUS (a score greater or equal to 2) and 36 healthy controls (a score less than 2) [Fig 1A], with the distribution of scores shown in Fig 1B. From the same group of calves, 20 cases of BRD were identified by WHS (a score greater or equal to 5) and 45 controls (a score less than 5) [Fig 1C]. Overall, 22 calves were assigned to controls as negative cases of BRD (both TUS and WHS negative), 24 assigned to a case of BRD diagnosed by TUS only (TUS positive and WHS negative), 14 BRD cases diagnosed by WHS only (TUS negative and WHS positive), and 6 animals diagnosed with BRD by both techniques (TUS and WHS positive) [Fig 1D]. Hematological analysis of white blood cell, lymphocyte, neutrophil, monocyte, eosinophil, and basophil cell counts circulating in blood was revealed using a linear regression model correcting for both age and sex no significant differences between controls and TUS positive calves or WHS positive calves (Fig 2A and 2B). Results showed that age had a significant positive association with basophil counts (*p* = 0.0020).

### Similar innate immune signalling genes expression patterns in 4-hour and 24-hour unstimulated whole blood from control and BRD diagnosed calves

Next, multiplex gene expression analysis of 48 genes was carried out of the calves chosen based on their TUS positivity. Gene expression was measured in 4 hour and 24-hour unstimulated whole blood using *ImmunoChek* assay. Controls selected had a TUS score of zero while BRD cases selected had a TUS score of 3–4. Firstly, principal component analysis (PCA) of 4 hour and 24-hour unstimulated gene expression revealed no distinct clustering of BRD cases from controls at both timepoints (S2A and S2B Fig). Hierarchical clustering of unstimulated gene expression revealed a cluster of genes at 4-hours (*IL6R*, *IL6ST*, *IFNAR*, *RXRA and IFNGR1*) with similar high expression patterns and 24-hours (*TGFB1*, *STAT3*, *CASP4*, *STAT2*, *IL18* and *RXRA*) with similar high expression patterns (S2C and S2D Fig). However, differential gene expression analysis revealed one gene significantly increased in BRD cases compared to controls at 4 hours (*IFNGR1*) and three significantly increased at 24-hours (*RXRA*, *IL18* and *IFNAR1*), with no significant differentially expressed genes found after *p*-value correction.

### Differential gene expression patterns between control and BRD diagnosed calves is preferentially in response to TLR1/2 ligand Pam3CSK4 after 24-hour stimulation

Gene expression analysis of 48 genes was carried out on 4 hour and 24-hour PAMP stimulated whole blood from TUS positive (n = 6) and control calves (n = 5), the same calves unstimulated gene expression analysis was completed. PCA revealed no distinct clustering of BRD cases from controls at in response to all PAMPs at 4-hours (S3A Fig). Hierarchical clustering of log2 of fold change values relative to unstimulated gene expression revealed increased gene expression of chemokines, IL-1 family genes, inflammasome complex and vitamin D pathway in response to 4-hour PAMP stimulation at 4 hours (S3B Fig). Differential gene expression analysis between BRD calves and controls found one gene significantly decreased in response to LPS at 4 hours (*IFNGR1*), with no significant differential expression found after p-value correction. In response to R848, one gene was significantly increased (*IFNA*), while three genes significantly decreased (*IL6ST*, *RXRA* and *TGFB1*). No significant differential expression in response to 4-hr PAMP stimulation was found after *p*-value correction. Adjusted *p*-values between BRD cases and controls are shown in S2 Table.

**Fig 3:**
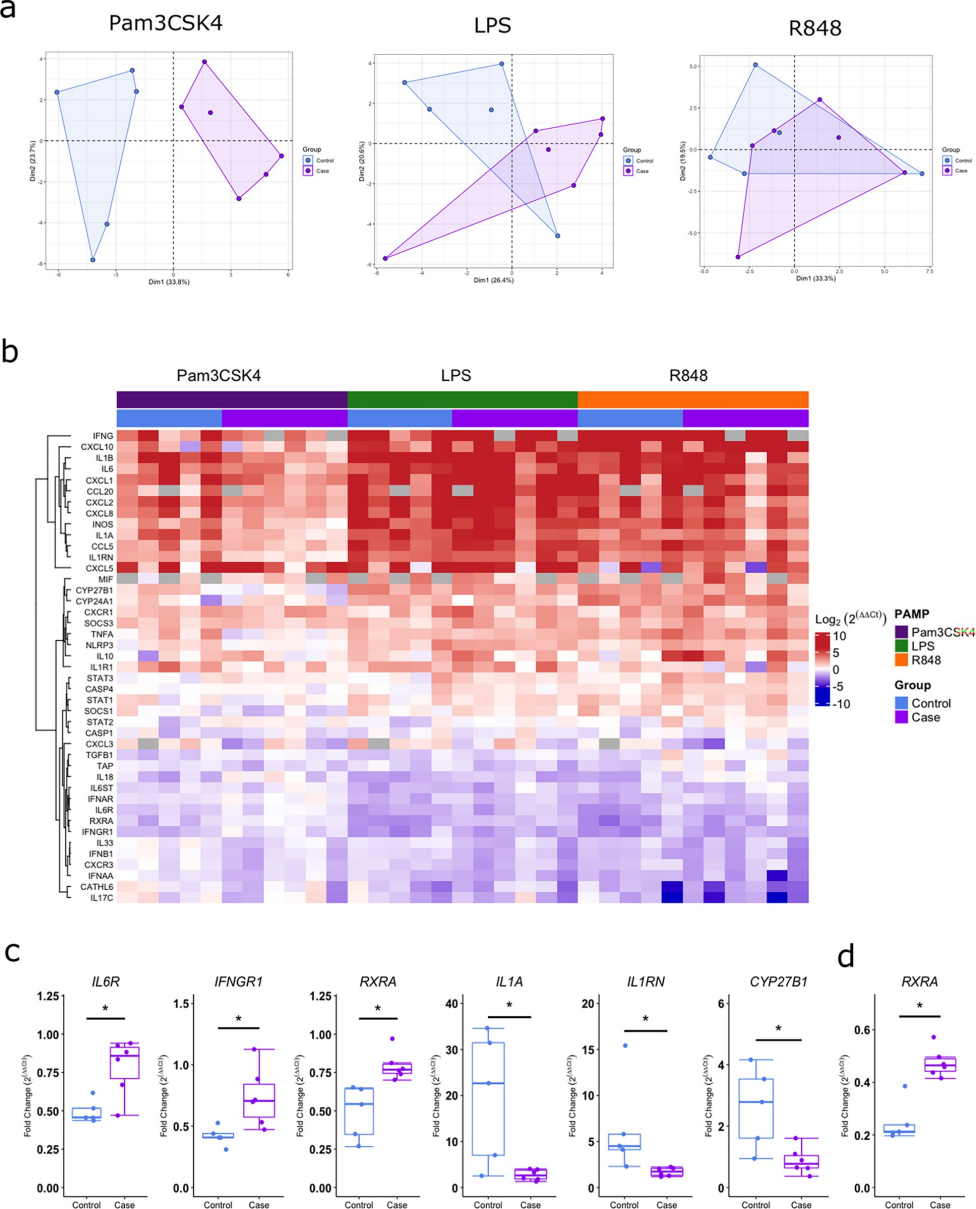
IL-6 responses to PAMPs as potential biomarkers of TUS positivity. ROC curves (x = false positives, y = true positives) of IL-6 protein concentrations in response to LPS, Pam3CSK4 and R848 in control and TUS positive calves. AUC shows an 0.68, 0.83 and 0.66 probability and Youden index shows an optimum accuracy of 47.0%, 66.4% and 30.7% for IL-6 protein concentrations in response to LPS, Pam3CSK4 and R848 respectively in diagnosing BRD calves with pathological lung lesions determined by thoracic lung ultrasonography scoring.

In 24-hour PAMP stimulated samples, PCA revealed distinct clustering of gene expression in response to Pam3CSK4 between BRD cases and controls (Fig 3A). Distinct clustering was not found in response to LPS or R848 (Fig 3A). Hierarchical clustering and heatmap representation of log2 of fold change values relative to unstimulated gene expression revealed increased gene expression of chemokines, IL-1 family genes, inflammasome complex and vitamin D pathway in response to 24-hour PAMP stimulation (Fig 3B). Furthermore, a decreased pattern of expression of the cluster of upregulated genes in response to PAMP stimulations is observed in response to Pam3CSK4 in BRD cases (Fig 3B). Differential gene expression analysis between BRD calves and controls found 7 genes significantly increased (*IL18*, *TGFB1*, *IL6R*, *IFNGR1*, *IFNAR*, *STAT3* and *RXRA*) and 6 significantly decreased (*TNFA*, *IL1B*, *IL1A*, *IL1RN*, *CXCL2* and *CYP27B1*) in response to Pam3CSK4 for 24 hours. After *p*-value correction, *IL6R*, *IFNGR1* and *RXRA* remained significantly increased and *IL1A*, *IL1RN* and *CYP27B1* significantly decreased in BRD cases, with gene expression fold changes relative to unstimulated shown in Fig 3C. In response to LPS, one gene was significantly increased (*RXRA*) and 2 significantly decreased (*IL33* and *IFNB1*). After *p*-value correction, *RXRA* remained significantly increased with the gene expression fold change relative to unstimulated shown in Fig 3D. In response to R848, one gene was found significantly increased (*IL18)*, with no significant differential expression found after *p*-value correction. Adjusted *p*-values between BRD cases and controls are shown in S2 Table.

### Impaired IL-1 and inflammasome and chemokine gene expression patterns in response to Pam3CSK4

**Fig 4:**
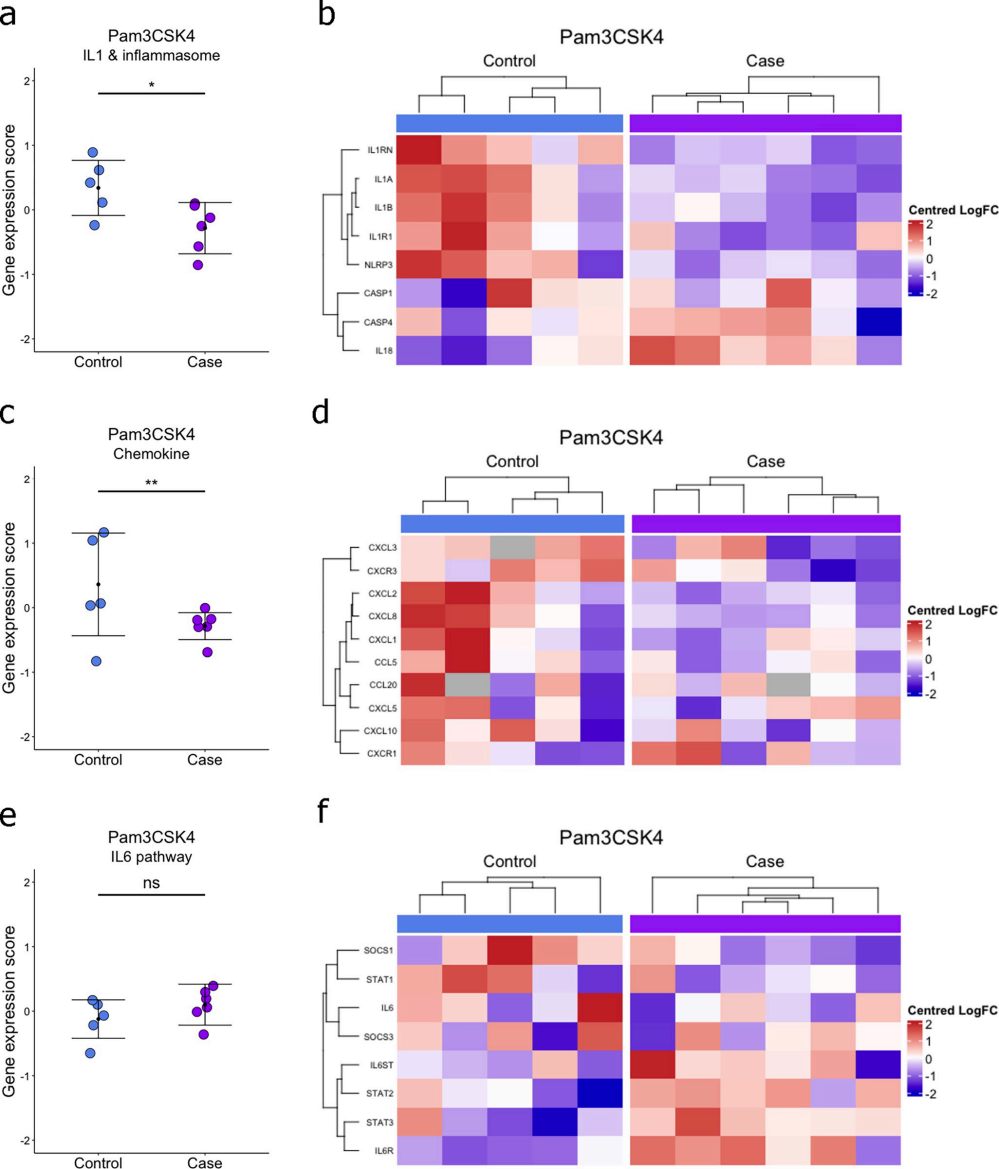
Differential expression of innate immune genes in response to PAMPs in controls and BRD diagnosed calves is in response to TLR1/2 ligand Pam3CSK4. (a) PCA plot, and (b) hierarchical clustering and heatmap of the Log2 fold change in expression relative to unstimulated samples of the innate immune genes in response to LPS, Pam3CSK4 and R848 24-hour whole blood stimulation in control (n = 5) and BRD diagnosed (Case) (n = 6) calves aged 2–8 weeks old. (c-d) Boxplots of the fold change in expression relative to unstimulated samples of significant genes found between control (n = 5) and BRD diagnosed (Case) (n = 6) in response to (c) Pam3CSK4 and (d) LPS after p value adjustment. p adjusted<0.1*. The box represents the interquartile range, the whiskers the upper and lower limits and the dots representing each calf. T-tests were used to calculate significant differences with p-values adjusted by FDR method. *p-adjusted <0.1.

Gene expression scores were calculated for IL-1 and inflammasome genes (*IL1A*, *IL1B*, *IL18*, *IL1RN*, *IL1R1*, *NLRP3*, *CASP1* and *CASP4*), chemokine genes (*CCL20*, *CCL5*, *CXCL1*, *CXCL10*, *CXCL2*, *CXCL3*, *CXCL5*, *CXCL8*, *CXCR1* and *CXCR3*) and IL-6 signalling genes (*IL-6*, *IL6R*, *IL6ST*, *STAT1*, *STAT2*, *STAT3*, *SOCS1* and *SOCS3*) for calf responses to PAMPs. BRD calves were found to have a significantly decreased IL-1 and inflammasome gene expression score in response to Pam3CSK4 (Fig 4A). No significant differences in IL-1 and inflammasome gene expression scores were found in response to LPS and R848. Hierarchical clustering with centered and scaled gene expression fold changes shows a decreased IL-1 and inflammasome gene expression in BRD calves (Fig 4B). Conversely, *IL18* and *CASP4* individually showed similar increased expression pattern in BRD calves (Fig 4B). BRD calves were also found to have a significantly decreased chemokine gene expression score in response to Pam3CSK4 (Fig 4C). No significant differences were found in chemokine gene expression scores in response to LPS and R848. Hierarchical clustering with centered and scaled gene expression fold changes shows a decreased chemokine gene expression in BRD calves (Fig 4D). No significant differences in gene expression scores for IL-6 signalling in response to PAMPs, including Pam3CSK4 (Fig 4E). However, hierarchical clustering with centered and scaled gene expression fold changes in IL-6 signalling genes in response to Pam3CSK4 showed clustering of *IL6ST*, *IL6R*, *STAT1* and *STAT3* that had increased expression patterns and clustering of *SOCS1* and *STAT2* which has decreased expression patterns in BRD calves (Fig 4F).

### Similar IL-6, IL-1β and IL-8 protein expression in 24-hour unstimulated whole blood from control and calves with BRD

**Fig 5:**
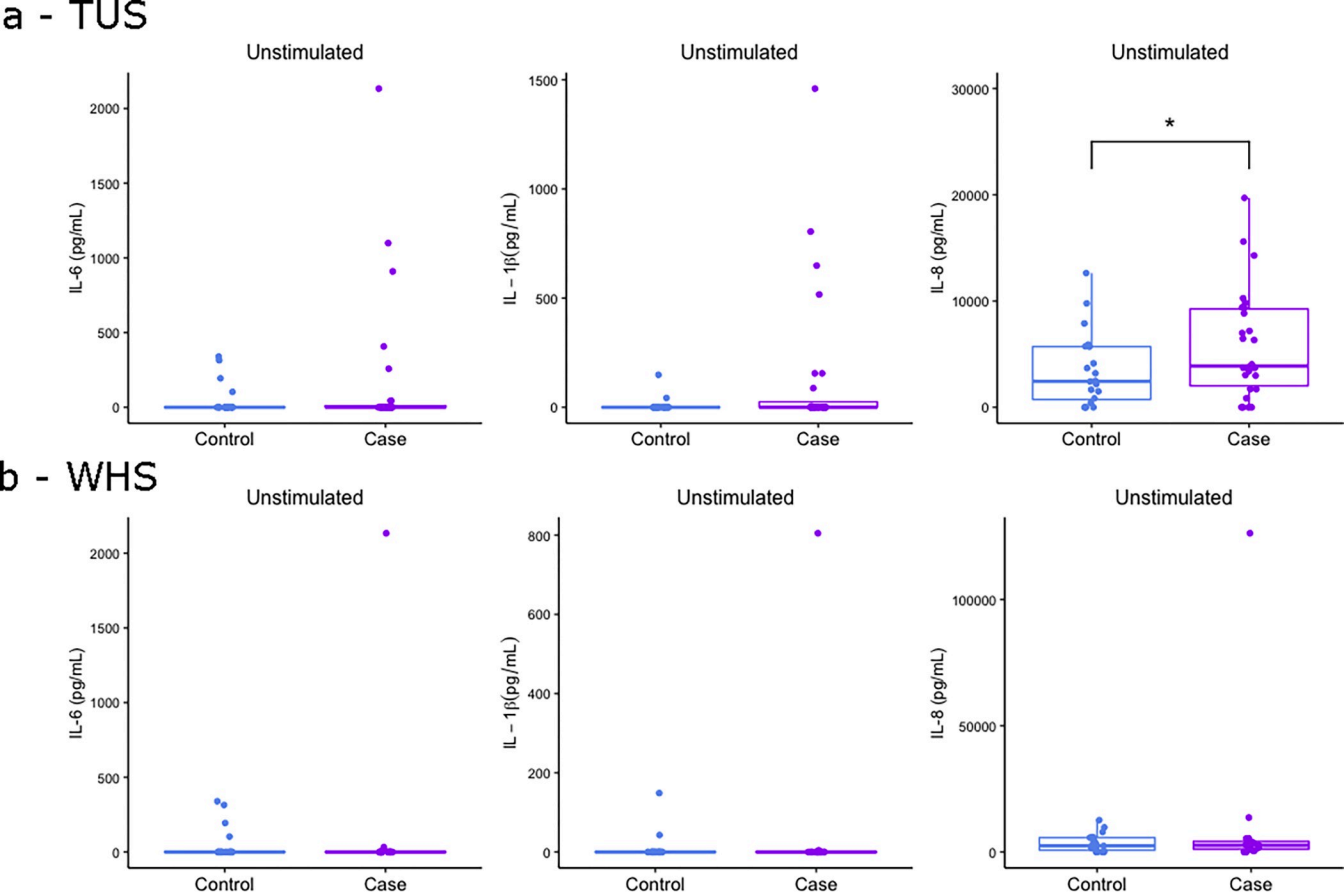
Decreased IL-1 and inflammasome, and chemokine gene expression pattens in response to Pam3CSK4 in BRD diagnosed calves. (a) IL-1 and inflammasome, (c) chemokine and (e) IL-6 pathway gene expression z-scores in response to Pam3CSK4 in whole blood of controls and BRD diagnosed calves aged 2–8 weeks old. Error bars represent mean +/- standard deviation. T-tests were used to test for significant differences between gene expression scores. *p<0.05, **p<0.01. (b) IL-1 and inflammasome, (d) chemokine and (f) IL-6 pathway hierarchical clustering and heatmap of centred and scaled per unit variance LogFC gene expression relative to unstimulated samples in BRD blood of controls and BRD diagnosed calves aged 2–8 weeks old.

A linear regression model integrating both age and sex, found significantly increased 24 hour unstimulated IL-8 levels in TUS positive calves compared to controls, but not for IL-1β and IL-6. Protein levels were undetectable between controls, TUS positive and WHS positive calves were found for IL-6 and IL-1β, with 49 out of 61 and 51 out of 61 calves having no detectable levels of cytokine respectively (Fig 5A and 5B). However, outlier analysis (Tukey analysis) revealed the same three TUS positive calves displayed the highest 24-hour unstimulated IL-6, IL-1β and IL-8 (Fig 5A). Similarly, outlier analysis revealed that one WHS positive calf displayed the highest 24-hour unstimulated IL-6, IL-1β and IL-8 (Fig 5B).

### IL-6 hyper-response to PAMP stimulated whole blood in calves with BRD

**Fig 6:**
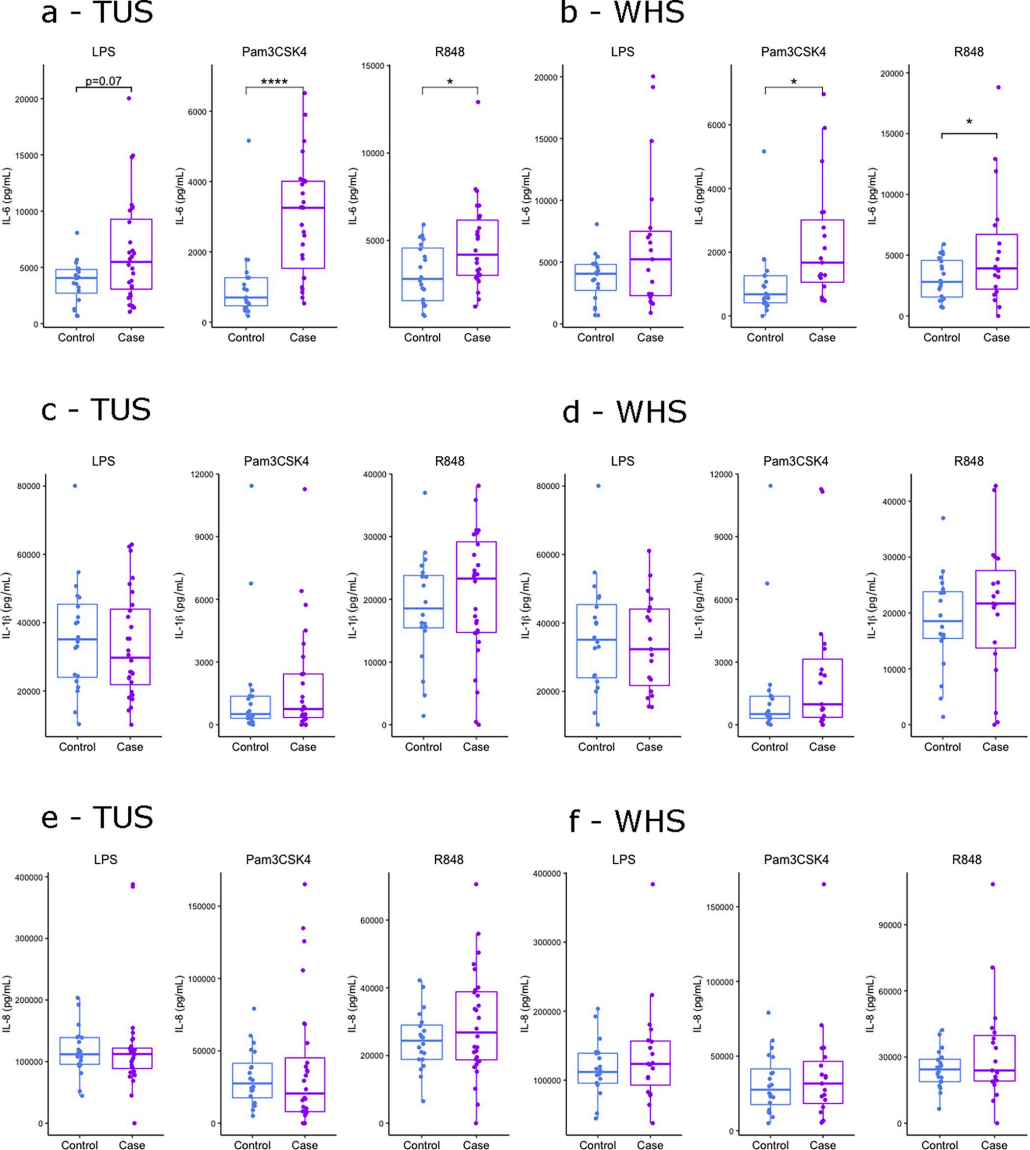
Elevated levels of IL-8 protein expression in unstimulated whole blood of BRD diagnosed calves. Boxplots of IL-6, IL-1β and IL-8 protein expression in 24-hour unstimulated whole blood of calves that were controls and (a) TUS positive and (b) WHS positive. The box represents the interquartile range, the whiskers the upper and lower limits and the dots representing each calf with any calves outside the upper and lower limits considered outliers (Tukey outlier analysis). T tests were used to test for significant differences between cases and controls. *p<0.05.

Protein expression of cytokines IL-1β, IL-6 and IL-8 were measured by ELISA in response to LPS, Pam3CSK4 and R848 in whole blood of TUS positive, WHS positive and controls calves. A linear regression model integrating age and sex found IL-6 significantly elevated in response to Pam3CSK4 and R848, and close to significantly increased in response to LPS (p = 0.07) in TUS positive calves compared to controls. TLR1/2 ligand Pam3CSK4 induced the most differential response (Fig 6A). IL-6 was also significantly increased in response to Pam3CSK4 and R848 in WHS positive calves (Fig 6B). However, this was not to the same extent observed in TUS positive calves in response to Pam3CSK4. Furthermore, no significant differences were found in IL-6 in response to LPS in WHS positive calves (Fig 6B). Similar levels of IL-1β in response to all three PAMPs in TUS and WHS positive calves compared to controls (Fig 6C and 6D). Similarly, levels of IL-8 remained unchanged in response to all three PAMPs in TUS and WHS positive calves compared to controls (Fig 6E and 6F).

### ROC curve analysis reveals Pam3CSK4 IL-6 responses as a potential biomarker of BRD

**Fig 7:**
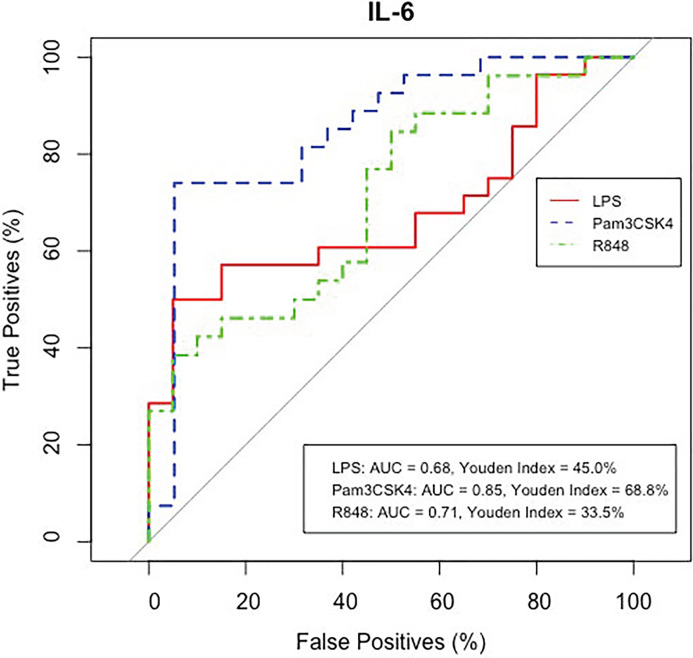
Elevated IL-6 response to PAMP stimulated whole blood of calves diagnosed with BRD by TUS. Boxplots of (a-b) IL-6, (c-d) IL-1β and (e-f) IL-8 protein expression in 24-hour stimulated whole blood with PAMPs LPS, Pam3CSK4 and R848 in 2–8 weeks old control, (a, c and e) TUS positive, and (b, d and f) WHS positive calves. The box represents the interquartile range, the whiskers the upper and lower limits and the dots representing each calf with any calves outside the upper and lower limits considered outliers (Tukey outlier analysis). T tests were used to test for significant differences between cases and controls. *p<0.05, **p<0.01, ***p<0.001, ****p<0.0001.

A receiver operator characteristic (ROC) curve was used to determine the efficacy of BRD diagnosis based on IL-6 responses to PAMPs in both TUS positive and healthy control calves (Fig 7). In response to LPS and R848, IL-6 had an area under the curve (AUC) of 0.68 and 0.71, and Youden Index (YI) of 45.0% and 33.5% respectively in determining BRD positive calves based on TUS. At the optimum IL-6 protein concentration threshold of <5679.3 pg/mL, the true and false positive rate for LPS was 50% and 5%, respectively. Similarly, the true and false positive rate for R848 was 38.5% and 5% respectively, at an IL-6 protein concentration threshold <5284.9 pg/mLs. IL-6 response to Pam3CSK4 was identified as the most sensitive and specific biomarker with a AUC of 0.85 in diagnosing BRD (based on TUS) and YI of 68.8% for optimal classification of cases and controls. Furthermore, at the optimum level of <1780.3 pg/mL IL-6, the true and false positive rates were 74.1% and 5.2% false positive rate, respectively (Fig 7).

## Discussion

BRD is one of the most significant causes of mortality in early life of calves, with a weakened immune system having a high impact on disease susceptibility. Vaccination has not significantly reduced disease burden in young calves, implicating host innate immunity as a key factor in both susceptibility to and pathology of BRD [5]. However, the early innate immune responses associated with pathology remain largely uncharacterized, particularly in naturally infected cattle. In this study we applied a standardized approach using a whole blood stimulation assay to carry out high throughput innate immune phenotyping in calves across 9 farms diagnosed with BRD using two assessment methods: WHS and TUS. Lack of concordance in diagnosis was observed between techniques indicating that that most WHS positive calves may not have lower respiratory tract infections, and therefore a possible milder or less advanced stage of disease. Additionally, most TUS-positive calves likely have early-stage lung lesion development without hallmark signs of infection i.e., nasal discharge, high temperature and cough. This highlights the necessity for both methods to be used in conjunction for maximum sensitivity of BRD diagnosis.

In terms of host immunity, no changes were found in hematological profiles between BRD cases (diagnosed by TUS and/or WHS) and healthy controls. Other studies have reported significantly increased circulating neutrophils in both TUS positive and WHS positive BRD infected calves [28–30]. This might be explained by breed and/or age differences between cohorts, as in the mentioned study calves were 6–7 months old, whereas in the current study they were 2–8 weeks of age. Additionally, the calves in the current study may be in an earlier stage of disease in which circulating immune changes are not yet apparent. Interestingly, 24-hour unstimulated levels in whole blood of IL-8 were found increased in TUS positive calves in the current study, indicating potential as a sensitive biomarker for lung lesion development in calves and should be investigated further. Although previous research shows elevated pro-inflammatory systemic response in BRD infected calves, in this study no pronounced indications of a baseline inflammatory profile was apparent at either gene expression or protein levels [31, 32]. Therefore, in conjunction with the hematological results, the lack of gene expression changes found in unstimulated cells, results suggest that immune stimulation may be required to identify early-stage disease biomarkers. It must be noted however, that immune profiles from *in vitro* unstimulated whole blood is not a fully accurate reflection of that detectable in serum and therefore comparisons should be carefully interpreted.

The majority of significant molecular changes detected in BRD infected calves after PAMP stimulation were found in response to the TLR1/2 activator Pam3CSK4, including an elevated IL-6 response and decreased IL-1 and inflammasome pathway and chemokine gene expression profile. TLR1/2 is activated by lipids, lipopeptides and peptidoglycans found typically in high abundance on Gram-positive bacteria. This finding suggests the potential presence Gram-positive bacterial infection which should be evaluated in future studies. Also, *Mycoplasma bovis* is commonly found in calves with pneumonia, and *in vitro* studies have shown lipids derived from this bacteria activate the pro-inflammatory response in embryonic bovine lung cells in a TLR2 activation dependent manner [33]. In addition, bovine mammary epithelial cells stimulated with *Mycoplasma bovis* showed upregulation of both TLR2 and IL-6 expression [34]. Studies have found interrelationships where *Mycoplasma bovis* infection can increase the abundance of pneumonia when present in conjunction with other pathogens including *M*. *haemolytica* [35]. Therefore, *Mycoplasma bovis* infection and perturbation of TLR1/2 signalling may be a possible mechanism by which homeostasis is disrupted during BRD pathology.

Interesting immune signalling genes found altered upon TLR1/2 stimulation including *IFNGR1*, the IFN-γ receptor 1 protein, which can modulate IFN-γ effects such as activation of Th1 immunity [36, 37]. Results also showed decreased activation of IL-1 and inflammasome pathway gene expression, which is essential for IL-1β secretion. In humans, inflammasome activation has been shown to be inhibited by TB infection [38]. However, the inflammasome responses during BRD infection has been largely uncharacterized until now. IL-1β secretion has been observed in blood of BRD infected cattle [18]. However, blockade of IL-1β does not improve infection outcomes [39]. Therefore, loss in ability to elicit inflammasome responses could be a mechanism of increased BRD pathology. Additionally, *IL1A* was found significantly decreased which can be increased in the inflammasome response, and is key in promoting inflammatory activity during BRD infection [31, 40]. Impaired chemokine responses were also observed, which identified another immune mechanism not previously characterized in BRD pathology. IL-8 has been previously reported as increased in blood of BRD infected animals [18]. IL-8 is critical for neutrophil recruitment and activation of their antimicrobial functions during BRD infection [41]. However, neutrophil recruitment can also increase immunopathology in BRD infected calves. Nevertheless, it is not clear if this impairment is an important change in the immune response to avoid increased pathological inflammation and severity of disease, or indicates immune suppression contributing to the development of severe disease [42, 43].

The defining feature of the immune response in BRD infected calves in this study, was a hyper-response in IL-6 protein expression to PAMP stimulation. This association was amplified in response to TLR1/2 stimulation in calves diagnosed with BRD by TUS. In addition, given the association was not as strong in WHS positive calves. Therefore, the hyper-IL-6 appears to be correlated to increased lower respiratory tract pathology which is being identified by TUS. A plausible explanation for this is the lung pathology in TUS positive calves is more likely being driven by bacterial infection associated with pneumonia. Therefore, this may be a more severe infection than in WHS positive calves, inducing more immune changes. Also, LPS and Pam3CSK4 activate TLRs known to be typically prominent in response to bacterial infection. The high induction of IL-6 in BRD infected calves is interesting as this cytokine has a documented strong association with respiratory disease in humans, with increased levels in serum positively associated with increased severity of COVID-19, respiratory syncytial virus, pneumonia and other chronic lung diseases [44–47]. No specific link has yet been made between IL-6 concentrations and the severity of BRD in cattle but concentrations have been previously shown as elevated in serum and tissue during established clinical disease [17, 18]. Upregulation of *IL6R* expression to TLR1/2 stimulation also further indicates BRD infection is altering TLR1/2 activation and via the IL-6 signalling pathway. Whether increased IL-6 responses reflect a protective response or promote pathology, remains an open question. IL-6 is critical for activation of CD4^+^ T-cells and Th2 responses during viral infections in humans [48, 49]. A SNP in *IL6* gene in humans is associated with decreased IL-6 responses and increased susceptibility to respiratory syncytial virus (RSV) [50]. However, IL-6 has been identified as the master regulator of the cytokine storm associated with severe disease in respiratory syndromes [51] and blocking the IL-6 pathway with immunotherapies have also shown to be an effective treatment against severe COVID-19 [52]. Therefore, this may be a mechanism by which BRD accentuates pathology. Future work could correlate the IL-6 hyper-responsiveness with adaptive immune phenotypes such as antibody titers to if it is activating an effective adaptive immune response.

Finally, IL-6 responses to TLR1/2 stimulation performed as an effective biomarker for BRD infection diagnosed by TUS, with an AUC of 0.85 and YI of 68.8%. Interestingly, we found stimulated IL-6 performed better in diagnosing BRD compared to another study assessing both serum IL-6 and C-reactive protein (CRP) for diagnosis of severe COVID-19, for which an AUC of 0.82 and 0.74 and a YI of 63% and 42% respectively [53]. Induced IFN-γ responses to TB antigens is commonly used in the diagnosis of TB in cattle, and therefore induced IL-6 responses to TLR1/2 stimulation should be further evaluated in its effectivity in BRD diagnosis [54]. Our study shows BRD infection increases the potential of the immune cells to produce IL-6, at early stage of infection, in advance of hematological changes and the development of pathology. Therefore, it may not only be an effective diagnostic biomarker but may be an early disease indicator that can enable early intervention strategies. Results herein are based on a matching sampling strategy of BRD infected and control calves whereas future work should assess the accuracy of prognostic classification based on IL-6 expression levels across all calves present on farms.

In conclusion, our study showed changes in innate immune responsiveness in BRD calves compared to controls. Altered immune responses were found preferentially in response to TLR1/2 ligand Pam3CSK4. This suggests a possible role for activation of this pathway in the pathology of BRD, as well as a potential involvement of bacteria such as *Mycoplasma bovis* in the BRD complex which remains less explored than other pathogens. The specific innate immune mechanism found altered in BRD calves was increased IL-6. At a gene expression level, results showed chemokine and IL-1 and inflammasome responses are also impaired in BRD calves. Future work should explore if these mechanisms are functionally protective during infection or are dysfunctional mechanisms that will increase severity of disease, as it may lead to new immunotherapies for BRD treatment and prevention. Further investigation should also identify if this IL-6 hyper-response is reproducible in adult calves with BRD. Overall, we have highlighted a role for innate immune phenotyping in improving BRD diagnostics and prognostics, specifically a hyper-induced IL-6 phenotype, which may ultimately lead to earlier and more sensitive biomarkers for disease.

## Supporting information

S1 Fig Thoracic ultrasound image of healthy air and severely consolidated lung. a) Image of a thoracic ultrasound on a (a) healthy air-filled lung with reverberation artefact and no lesions present, and (b) a severely consolidated lung with lung lesion present.
(TIFF)

S2 Fig Gene expression of innate immune signalling genes in 4 hour and 24 hour unstimulated whole blood in control and BRD diagnosed calves. (a-b) PCA plot, and (c-d) Hierarchical clustering and heatmap of the expression of 44 innate immune signalling genes in 4 hour (a and c) and 24 hour (b and d) unstimulated whole blood of controls (n = 5) and BRD diagnosed (Cases)(n = 6) calves aged 2–8 weeks old.
(TIFF)

S3 Fig Expression of innate immune genes in response to 4-hr PAMP stimulation are similar between controls and BRD diagnosed calves. (a) PCA plot, and (b) hierarchical clustering and heatmap of the Log2 fold change in expression relative to unstimulated samples of the innate immune genes in response to LPS, Pam3CSK4 and R848 4 hour whole blood stimulation in control (n = 5) and BRD diagnosed (Case) (n = 6) calves aged 2–8 weeks old.
(TIFF)

S1 Table Calf health information. (XLSX)

S2 Table Raw and adjusted P values for gene expression analysis. (XLSX)

S3 Table Oligonucleotide primers for gene expression. (XLSX)
