# Correction: Hyper-induction of IL-6 after TLR1/2 stimulation in calves with bovine respiratory disease

**DOI:** 10.1371/journal.pone.0326136

**Published:** 2025-06-09

**Authors:** Cian Reid, John Donlon, Aude Remot, Emer Kennedy, Giovanna De Matteis, Cliona O’Farrelly, Conor McAloon, Kieran G. Meade

In the published version of this article [1], the figure legends for Figs 3–7 were published in the incorrect order. The authors have provided a corrected version of figures and captions here.

**Fig 3:**
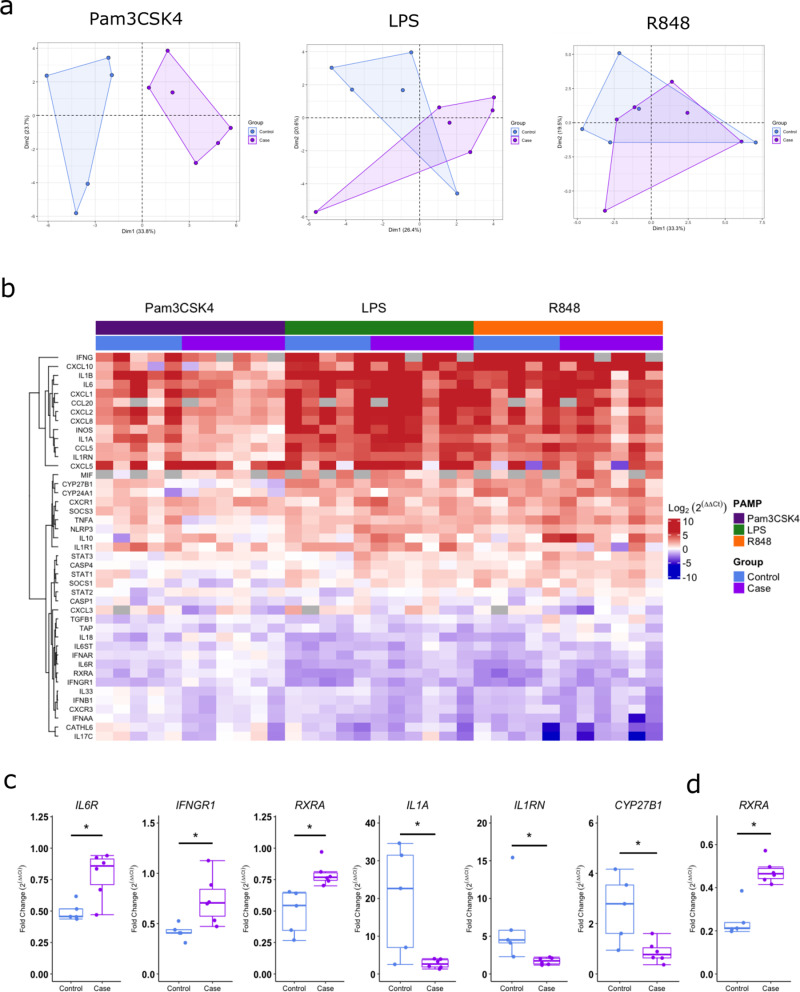
Differential expression of innate immune genes in response to PAMPs in controls and BRD diagnosed calves is in response to TLR1/2 ligand Pam3CSK4. (a) PCA plot, and (b) hierarchical clustering and heatmap of the Log2 fold change in expression relative to unstimulated samples of the innate immune genes in response to LPS, Pam3CSK4 and R848 24-hour whole blood stimulation in control (n = 5) and BRD diagnosed (Case) (n = 6) calves aged 2-8 weeks old. (c–d) Boxplots of the fold change in expression relative to unstimulated samples of significant genes found between control (n = 5) and BRD diagnosed (Case) (n = 6) in response to (c) Pam3CSK4 and (d) LPS after p value adjustment. p adjusted<0.1*. The box represents the interquartile range, the whiskers the upper and lower limits and the dots representing each calf. T-tests were used to calculate significant differences with p-values adjusted by FDR method. *p-adjusted <0.1.

**Fig 4:**
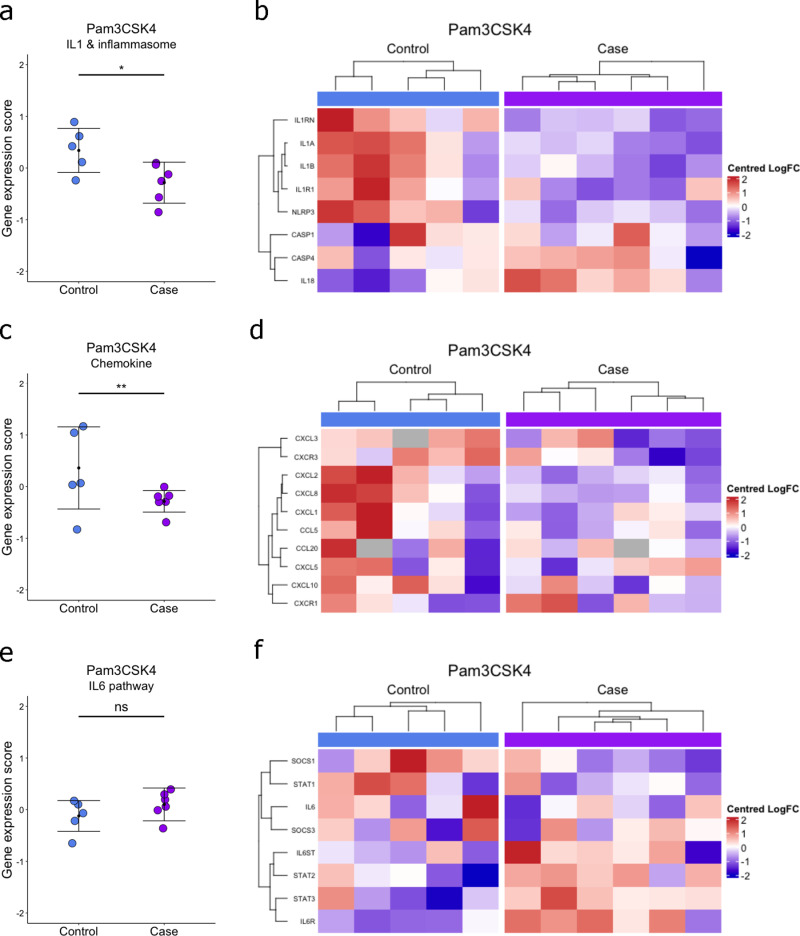
Decreased IL-1 and inflammasome, and chemokine gene expression pattens in response to Pam3CSK4 in BRD diagnosed calves. (a) IL-1 and inflammasome, (c) chemokine and (e) IL-6 pathway gene expression z-scores in response to Pam3CSK4 in whole blood of controls and BRD diagnosed calves aged 2-8 weeks old. Error bars represent mean ± standard deviation. T-tests were used to test for significant differences between gene expression scores. *p < 0.05, **p < 0.01. (b) IL-1 and inflammasome, (d) chemokine and (f) IL-6 pathway hierarchical clustering and heatmap of centred and scaled per unit variance LogFC gene expression relative to unstimulated samples in BRD blood of controls and BRD diagnosed calves aged 2–8 weeks old.

**Fig 5:**
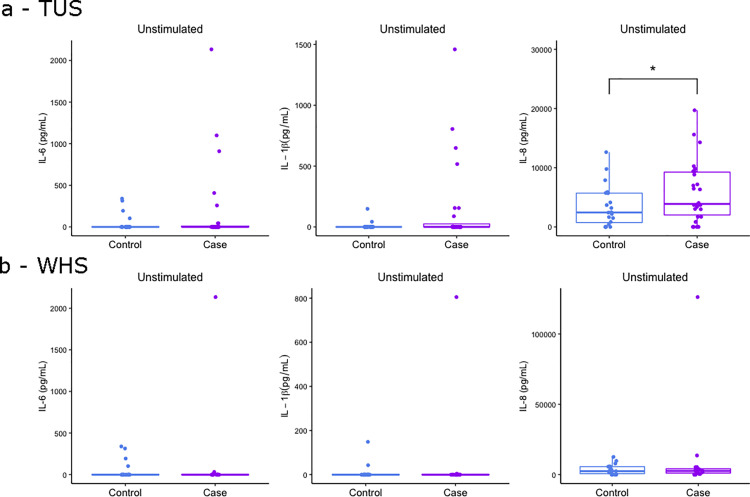
Elevated levels of IL-8 protein expression in unstimulated whole blood of BRD diagnosed calves. Boxplots of IL-6, IL-1β and IL-8 protein expression in 24-hour unstimulated whole blood of calves that were controls and (a) TUS positive and (b) WHS positive. The box represents the interquartile range, the whiskers the upper and lower limits and the dots representing each calf with any calves outside the upper and lower limits considered outliers (Tukey outlier analysis). T tests were used to test for significant differences between cases and controls. *p < 0.05.

**Fig 6:**
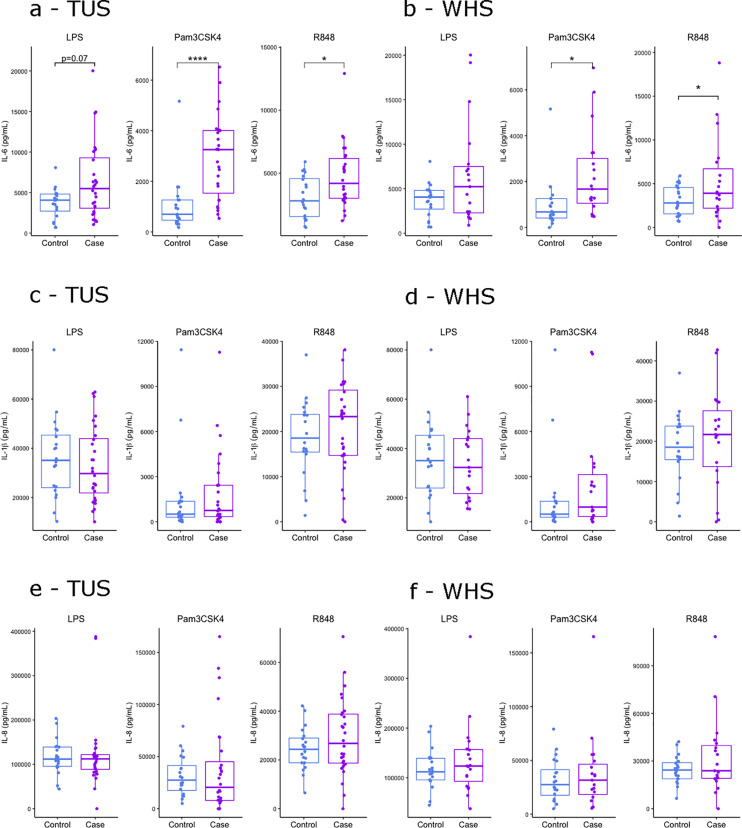
Elevated IL-6 response to PAMP stimulated whole blood of calves diagnosed with BRD by TUS. Boxplots of (a and b) IL-6, (c and d) IL-1β and (e and f) IL-8 protein expression in 24-hour stimulated whole blood with PAMPs LPS, Pam3CSK4 and R848 in 2-8 weeks old control, (a, c and e) TUS positive, and (b, d and f) WHS positive calves. The box represents the interquartile range, the whiskers the upper and lower limits and the dots representing each calf with any calves outside the upper and lower limits considered outliers (Tukey outlier analysis). T tests were used to test for significant differences between cases and controls. *p < 0.05, **p < 0.01, ***p < 0.001, ****p < 0.0001.

**Fig 7:**
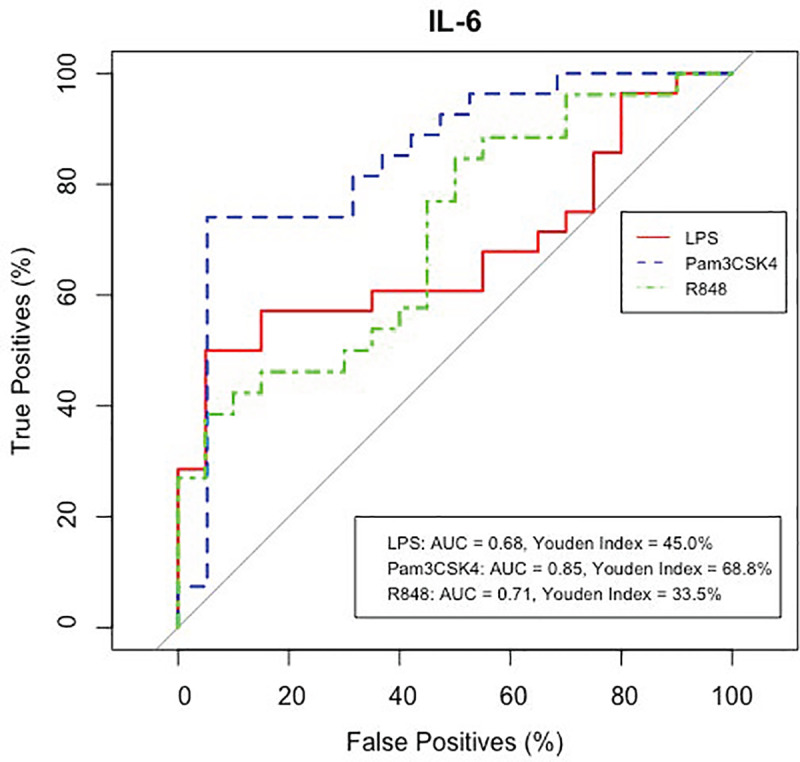
IL-6 responses to PAMPs as potential biomarkers of TUS positivity. ROC curves (x = false positives, y = true positives) of IL-6 protein concentrations in response to LPS, Pam3CSK4 and R848 in control and TUS positive calves. AUC shows an 0.68, 0.83 and 0.66 probability and Youden index shows an optimum accuracy of 47.0%, 66.4% and 30.7% for IL-6 protein concentrations in response to LPS, Pam3CSK4 and R848 respectively in diagnosing BRD calves with pathological lung lesions determined by thoracic lung ultrasonography scoring.
