# Supplementary figures and images for: Hyper-induction of IL-6 after TLR1/2 stimulation in calves with bovine respiratory disease
Source: PLoS One. 2024 Nov 14;19(11):e0309964. doi: 10.1371/journal.pone.0309964 (PMC11563416; doi:10.1371/journal.pone.0309964)

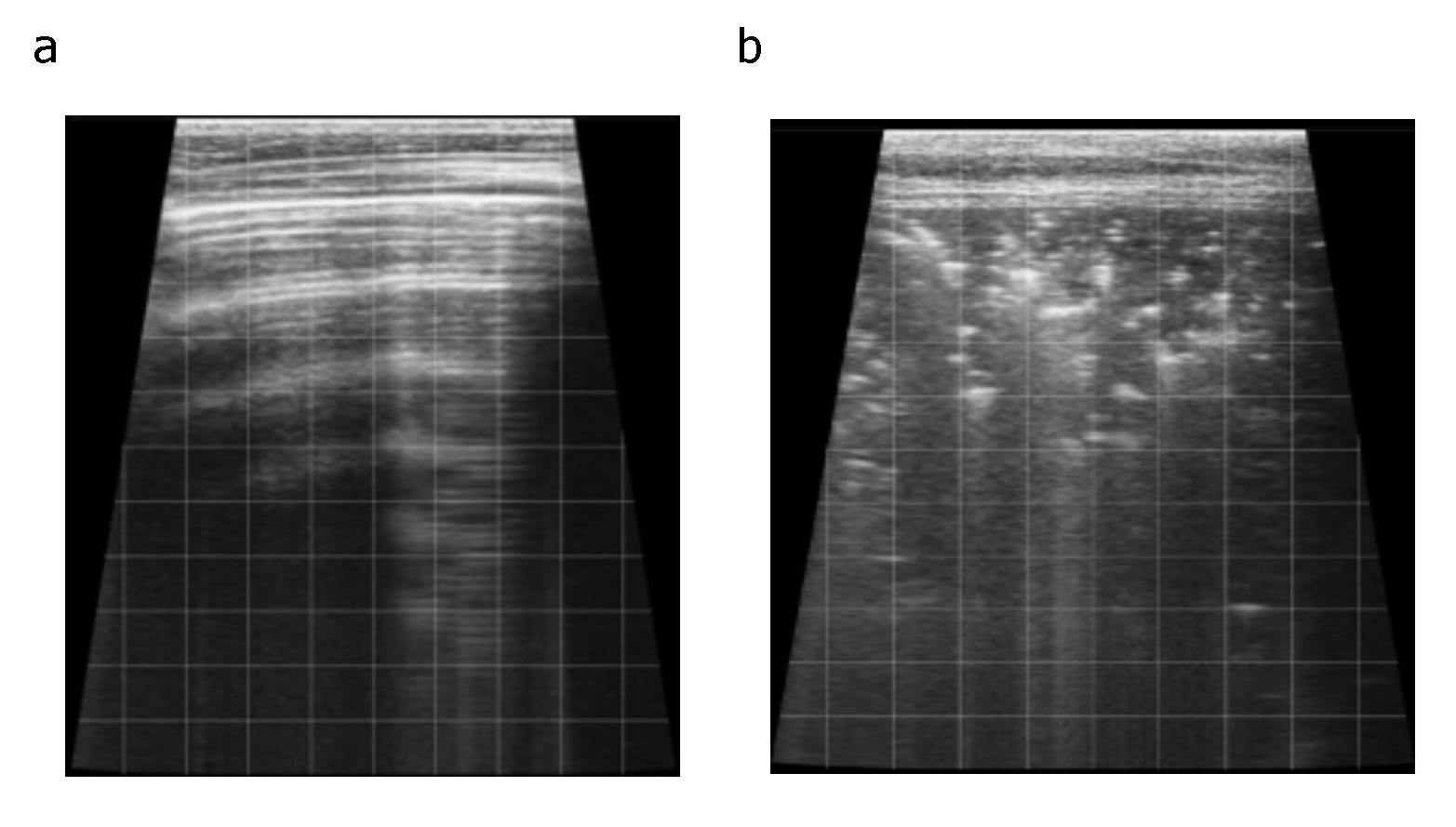

Supplement: S1 Fig — a) Image of a thoracic ultrasound on a (a) healthy air-filled lung with reverberation artefact and no lesions present, and (b) a severely consolidated lung with lung lesion present. (TIFF) [file pone.0309964.s001.tiff]

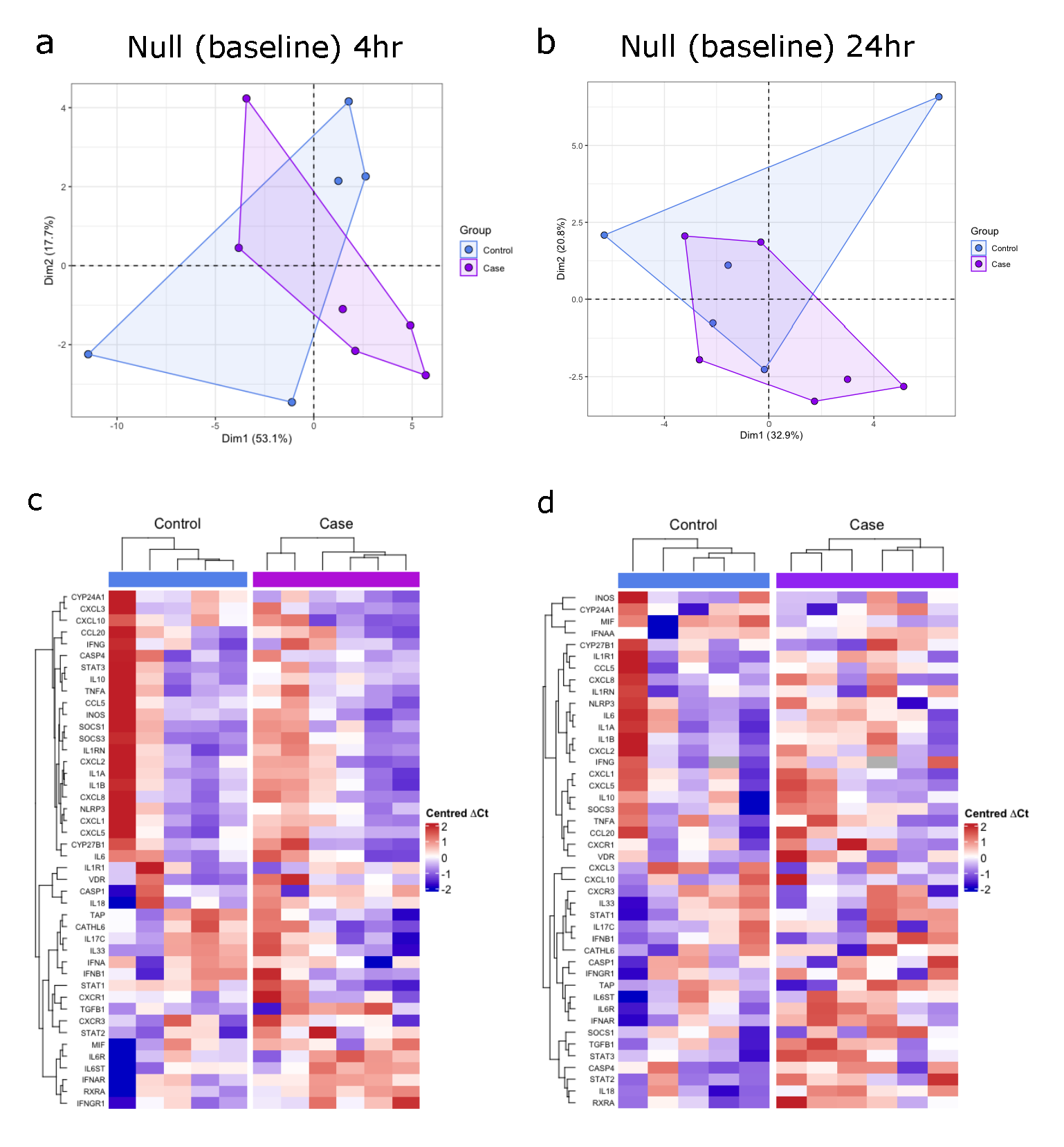

Supplement: S2 Fig — (a-b) PCA plot, and (c-d) Hierarchical clustering and heatmap of the expression of 44 innate immune signalling genes in 4 hour (a and c) and 24 hour (b and d) unstimulated whole blood of controls (n = 5) and BRD diagnosed (Cases)(n = 6) calves aged 2–8 weeks old. (TIFF) [file pone.0309964.s002.tiff]

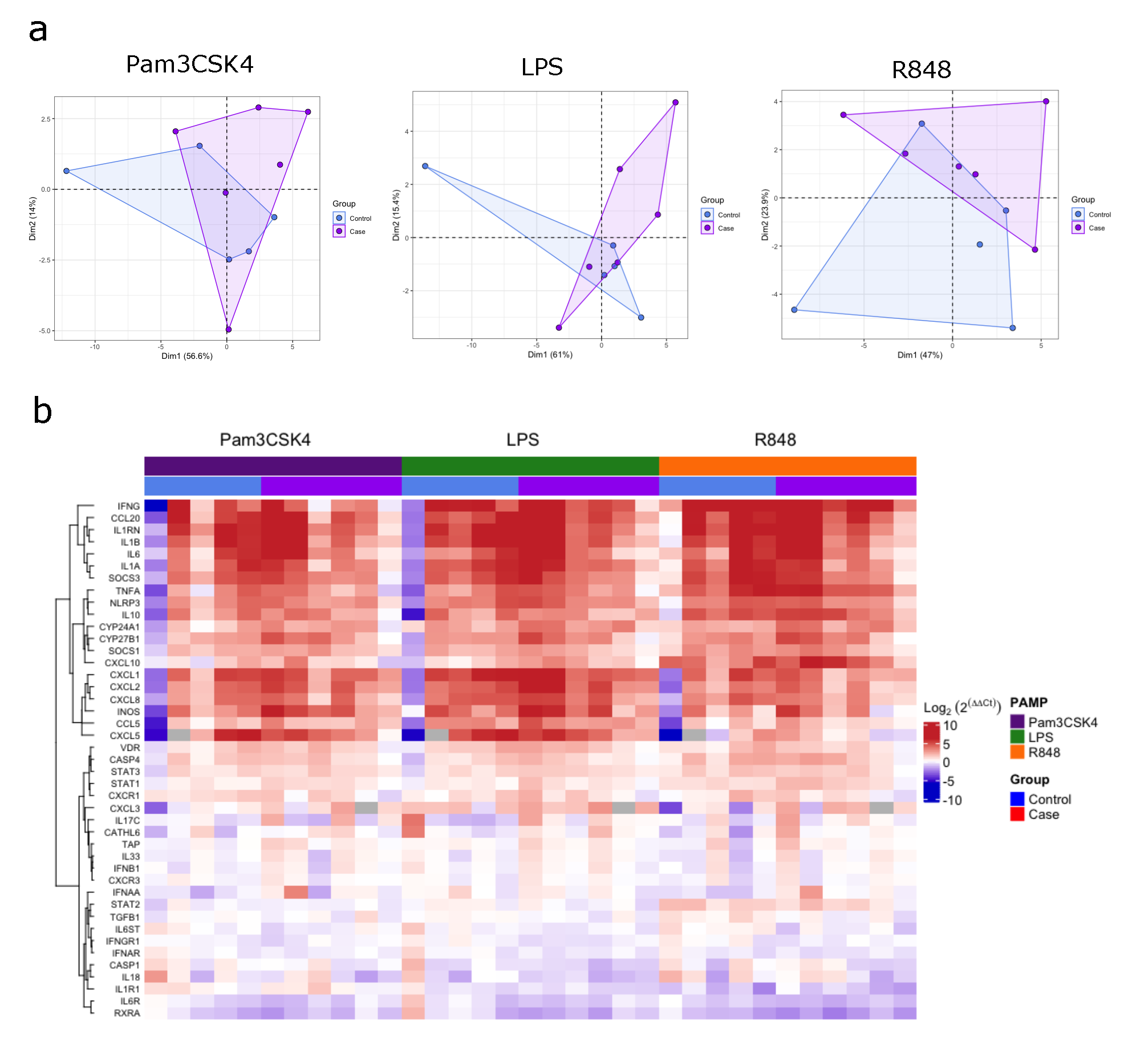

Supplement: S3 Fig — (a) PCA plot, and (b) hierarchical clustering and heatmap of the Log2 fold change in expression relative to unstimulated samples of the innate immune genes in response to LPS, Pam3CSK4 and R848 4 hour whole blood stimulation in control (n = 5) and BRD diagnosed (Case) (n = 6) calves aged 2–8 weeks old. (TIFF) [file pone.0309964.s003.tiff]
